# Pain Management in a Model of Interstitial Cystitis/Bladder Pain Syndrome by a Vaccinal Strategy

**DOI:** 10.3389/fpain.2021.642706

**Published:** 2021-03-08

**Authors:** Céline Augé, Lilian Basso, Catherine Blanpied, Nathalie Vergnolle, Xavier Gamé, Sophie Chabot, Philippe Lluel, Gilles Dietrich

**Affiliations:** ^1^Urosphere, Department of Pain and Inflammation, Toulouse, France; ^2^IRSD, Université de Toulouse, INSERM, INRA, ENVT, UPS, Toulouse, France; ^3^Department of Physiology and Pharmacology, Faculty of Medicine, University of Calgary, Calgary, AB, Canada; ^4^Urology Department, Rangueil University Hospital, Toulouse, France; ^5^INSERM, I2MC-U1048, CHU Rangueil, Toulouse, France

**Keywords:** vaccination, T lymphocytes, opioid, bladder pain syndrome, cyclophosphamide, cystitis

## Abstract

Current analgesic treatments for Interstitial Cystitis*/*Bladder Pain Syndrome (IC/BPS) are limited. Here, we propose a novel antinociceptive strategy exploiting the opioid-mediated analgesic properties of T lymphocytes to relieve from bladder pain. In a chronic model of IC/BPS in rats, we show that a secondary T cell response against intravesically administered ovalbumin prevents from visceral pain in OVA-primed animals. The analgesic effect is associated with the recruitment of T lymphocytes within the inflamed mucosa and is reversed by naloxone-methiodide, a peripheral opioid receptor antagonist. Similarly, intravesical instillation of BCG or tetanus toxoid antigens in vaccinated rats protects from pain in the same model. We show opioid-dependent analgesic properties of local vaccine antigen recall in a preclinical rat model of chronic cystitis. Since BCG bladder instillation is regularly used in humans (as anticancer therapy), our results open it as a new therapeutic positioning for a pain management indication for IC/BPS patients.

## Introduction

Interstitial cystitis/bladder pain syndrome (IC/BPS) is a chronic disorder characterized by pelvic pain, pressure or discomfort perceived to be related to the urinary bladder accompanied by at least one other urinary symptom such as persistent urge to void or frequency of voiding (1). Although the etiology and pathogenesis of IC/BPS have not yet been elucidated, numerous hypotheses including defects of the urothelial barrier, autoimmunity and neurogenic disorder have been proposed (2).

A number of therapies have been proposed to treat IC/BPS but, to date, only pentosan polysulfate (Elmiron®) medication is approved by United States Food and Drug Administration (FDA). Pentosan polysulfate is an oral heparinoid that would induce the regeneration of the urothelial glycosaminoglycan layer. A meta-analysis concluded, however, to a significant pain improvement only in 20–37% of the patients (3). Intravesical treatment with dimethyl sulfoxide (DMSO) display anti-inflammatory and muscle relaxant effects but seems to be efficient only in patients with Hunner's ulcers who represent <7% of IC/PBS patients (4). Other treatments dedicated to alleviate IC/BPS-related vesical pain, including non-steroidal anti-inflammatory drugs (NSAIDs), the anticonvulsant gabapentin (5) or opioid drugs (6), are frequently used but their efficacy remains limited and cause undesirable side effects (7).

It is now widely accepted that T lymphocytes display analgesic activity dependent on their ability to produce and locally release endogenous opioids (8–16). We have previously demonstrated that this property requires their activation by antigens (12, 17). Because peripheral delivery of opioids is preferred to achieve analgesia, mainly because it avoids central side effects (18–22), we hypothesize that, as previously shown in intestinal inflammatory disorders (23), recruitment of opioid-producing T cells may represent an interesting strategy to alleviate pain in IC/BPS (24, 25). Therefore, a vaccine strategy that triggers both T cell recruitment and local opioid release appears as a promising therapeutic option in the pain management in IC/BPS.

In this study, we presented a new anti-nociceptive potency of vaccine strategy exploiting the analgesic properties of T lymphocytes.

## Materials and Methods

### Animals

Considering that IC/BPS in men is relatively unusual, experiments were performed on female animals. Six to seven weeks-old Sprague-Dawley female rats (Janvier Labs, Saint-Berthevin, France) were acclimatized to the laboratory conditions for at least 3 days before starting experiments. Two or three rats were housed in polysulfone type Sealsafe plus GR900 cages (Tecniplast, Lyon, France) on a bed of wood chips (1200, Souralit, Girona, Spain) with free access to food (Rodent Maintenance Diet A04/10 from Safe) and water (0.2 μm filtered water) *ad libitum*. Species-appropriate environmental enrichment (Aspen brick, Plexx, Uden, Netherlands) was added in the cages. Animals were maintained under artificial lighting between 7:00 am to 7:00 pm (12 h) in a controlled ambient temperature of 22 ± 2°C, and relative humidity at 55 ± 10%. All procedures were conducted in accordance with the Guide for the Care and Use of Laboratory Animals of the European Council Directive (2010/63/UE) and were approved by French Animal Ethical Committee (application number CEEA-122-2014-28 and APAFIS#16506-2018082411278474).

### Vaccination and Induction of Cystitis

**Figure 1:**
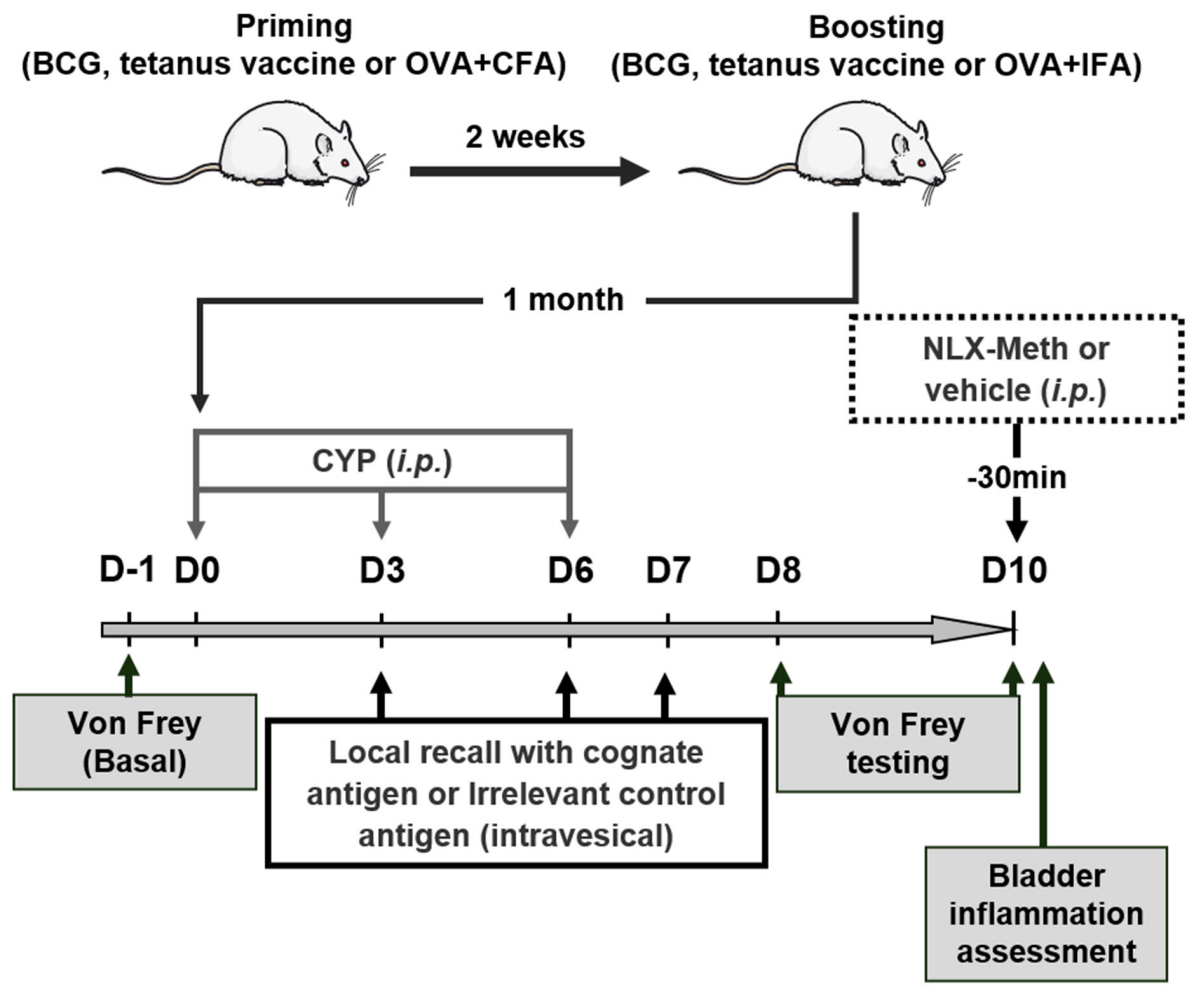
Experimental design. Rats were first vaccinated (immunized) 2 weeks apart. One month later, chronic cystitis was induced by three injections of CYP (40 mg/kg, i.p.) at days 0, 3, and 6. On day 3, 6, and 7 following the first injection of CYP, antigens previously used for vaccination (immunization) or irrelevant control antigens were intravesically instilled. Nociceptive response to mechanical stimuli (von Frey filaments) applied to vicinity of the bladder was performed 1 day before injecting CYP (D-1, basal bladder sensitivity) and at days 8 and 10 following the first injection of CYP to evaluate the effects of the vaccine strategy on CYP-induced chronic visceral pain. The role of peripheral opioid receptors in anti-nociceptive effects was investigated at day 10 using a general opioid receptor antagonist; the naloxone-methiodide (NLX-Meth, 10 mg/ml, i.p.), injected 30 min prior von Frey testing.

Animals were primed by subcutaneous injection of BCG vaccine (0.5 to 2 × 10^5^ UFC; 25 μL) (Statens Serum Institut, Copenhagen, Denmark), tetanus toxoid vaccine (40 IU/0.5 ml; 100 μl) (Sanofi Pasteur MSD SNC, Lyon, France) or OVA (200 μg) emulsified in complete Freund's adjuvant (CFA). Antigen recall was performed 2 weeks later with the same amounts of BCG vaccine, tetanus toxoid vaccine or OVA in incomplete Freund's adjuvant (IFA). One month after immunization, chronic cystitis was induced by 3 i.p. injections of CYP (Fisher-Scientific, Illkirch, France) 3 days apart (on days 0, 3 and 6) at 40 mg/kg as previously described (26). For antigen challenge, animals received intravesical instillation (0.8 ml) of either BCG (3 × 10^5^ to 5 × 10^6^ UFC) (Medac, Hamburg, Germany), a mixture of two tetanus immunodominant toxoid-derived peptides (IDKISDVSTIVPYIGPALNI and NNFTVSFWLRVPKVSASHLE) (50 μg/ml) (27, 28), OVA peptide 323–339 (ISQAVHAAHAEINEAGR) (5 mg/ml) (29, 30), OVA (1 mg/ml) or BSA (1 mg/ml) on days 3, 6, and 7. When indicated, rats were intraperitoneally injected with 200 μl naloxone-methiodide (10 mg/ml) at day 10 (11, 31). The experimental design is shown in Figure 1.

### Pain and Bladder Inflammation Assessment

Before the experiment starts, the animals were randomly assigned to treatment groups using the block method, which consists of distributing at least one animal per treatment or control in the same block. In addition, a different position in the von Frey chamber was assigned for animals of the same treatment group. Nociceptive response was assessed using von Frey filaments (26, 32). Scoring of nociceptive response was as follows: 0 = no response; 1 = reaction of the animal (e.g., retraction of the abdomen); 2 = reaction of the animal and change of position; 3 = reaction of the animal, change of position, licking of the area stimulated and/or vocalization. Macroscopic evaluation of bladder inflammation was performed as previously described (26). Bladder wall thickness was measured with an electronic caliper. Edema scoring was based on Gray et al. criteria (33) as follows: absent (0), mild (1), moderate (2) and severe (3).

### Immunochemistry

Bladders were fixed in 10% formalin and embedded in paraffin. For immunofluorescence staining of T lymphocytes five-micrometer tissue sections were incubated with1:50 diluted rabbit anti-CD3 monoclonal antibody (Clone SP7, Diagnostic-BioSystems, Pleasanton, CA) followed by Alexa Fluor 555-labeled goat anti-rabbit IgG antibody (Invitrogen, Carlsbad, CA); both for 1 h at room temperature. Nuclei were counterstained with 4′,6-Diamidino-2-Phenylindole (DAPI) fluorescent mounting medium (Vector laboratories Inc., Burlingame, CA). Images were acquired using a TCS SP8 confocal laser-scanning microscope with 20× objective (LEICA microsystems, Nanterre, France).

### Statistics

Data were analyzed with Prism 6 software (Graphpad, San-Diego, CA, USA). Before performing statistical tests, we determined whether the data were normally distributed and evaluated their variance. When normality condition was not met, non-parametric test was used (*i.e*., Mann-Whitney test). When variances were unequal, Welch's correction was applied. For nociceptive scores, a two-way analysis of variance (ANOVA) was used to determine whether there was a significant main difference among groups. When performing same measurement on a given animal at multiple time points (*i.e*., Basal, D8, D10), repeated measures (RM) were used. The accepted level of significance was *p* < 0.05. Data were expressed as mean ± SEM. The number of animal used is indicated in the legends.

## Results

### Evaluation of the Vaccine Strategy as Anti-nociceptive Treatment in an Interstitial Cystitis/Bladder Pain Syndrome Model

**Figure 2:**
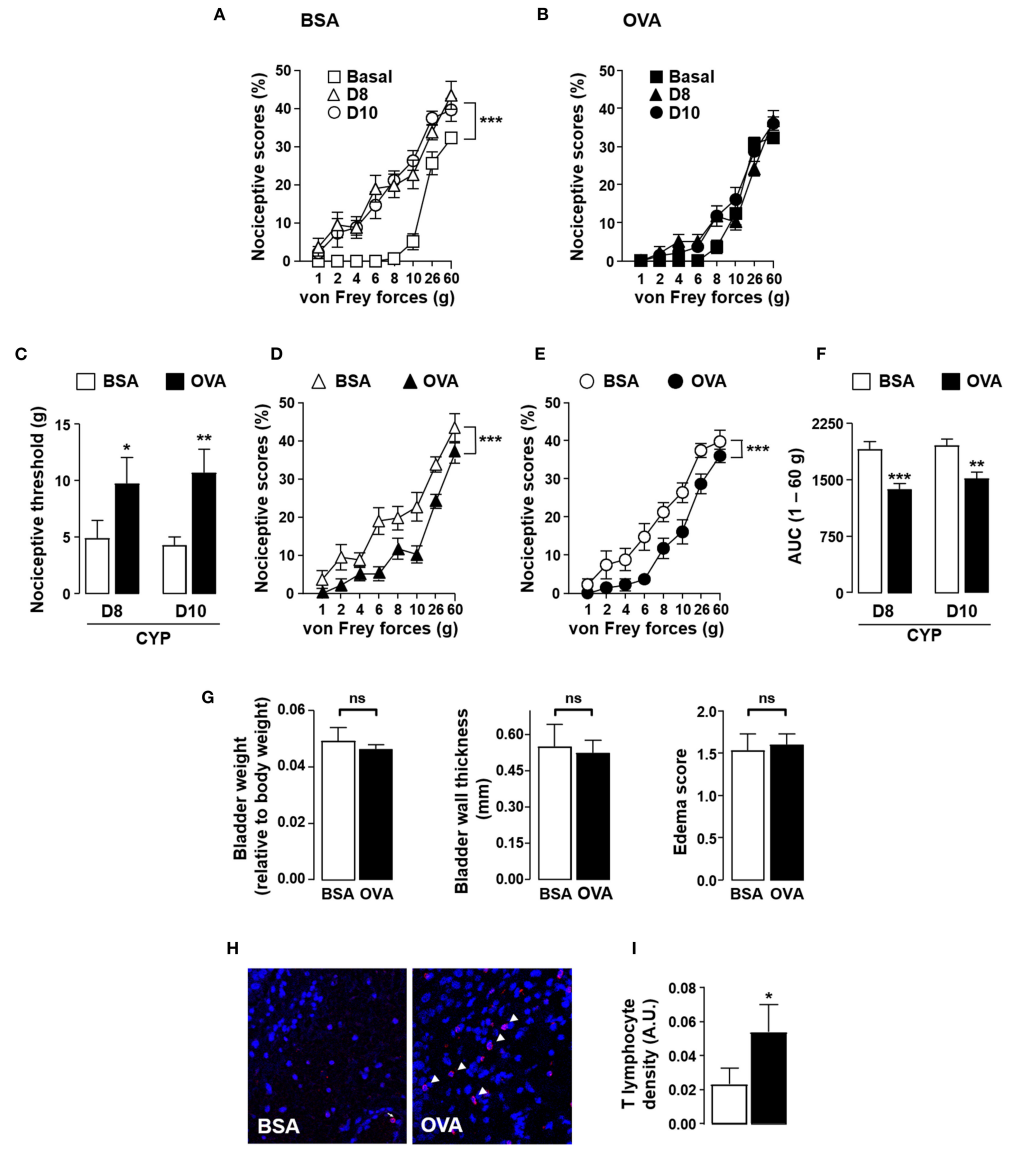
Instillation of OVA alleviates CYP-induced bladder pain in OVA-immunized rats. Rats were primed by subcutaneous injection of OVA emulsified in CFA and then boosted 2 weeks later with the same amount of OVA emulsified in IFA. One month after the last immunization, chronic cystitis was induced as described above. OVA-immunized rats were intravesically administered with either OVA or irrelevant control BSA antigen on days 3, 6 and 7. Von Frey testing was performed at days 8 and 10. **(A,B)** Nociceptive scores against von Frey forces from 1 to 60 g (hyperalgesia) before (basal, square) and at days 8 (triangles) and 10 (circles) after CYP treatment in OVA-primed rats intravesically instilled with BSA **(A)** or OVA **(B)**. **(C)** Nociceptive threshold defined as the von Frey force in grams at which a first score of at least one was obtained (allodynia) (*n* = 15 / group). **(D,E)** Nociceptive scores against von Frey forces at days 8 **(D)** and 10 **(E)** after CYP treatment in OVA-primed rats intravesically instilled with BSA or OVA. **(F)** Area under the curve (AUC) calculated by plotting individual nociceptive score against von Frey forces from 1 to 60 g (*n* = 15 / group). **(G)** Urinary bladder inflammation assessed at day 10 by bladder weight (left panel), wall thickness (middle panel) and edema scores (right panel). **(H)** Representative images of CD3 (T cells) immunostaining (red; arrowhead) in bladder from CYP-treated OVA-immunized rats intravesically instilled with either control antigen BSA (left panel) or OVA (right panel). Nuclei are counterstained with DAPI (blue). **(I)** Density of CD3^+^ T lymphocytes within bladder determined by quantifying anti-CD3 fluorescence relative to tissue surface delimited manually with DAPI staining (*n* = 11). Data represent mean values ± SEM. ns, no significant; *p < 0.05; **p < 0.01 and ***p < 0.001.

**Figure 3:**
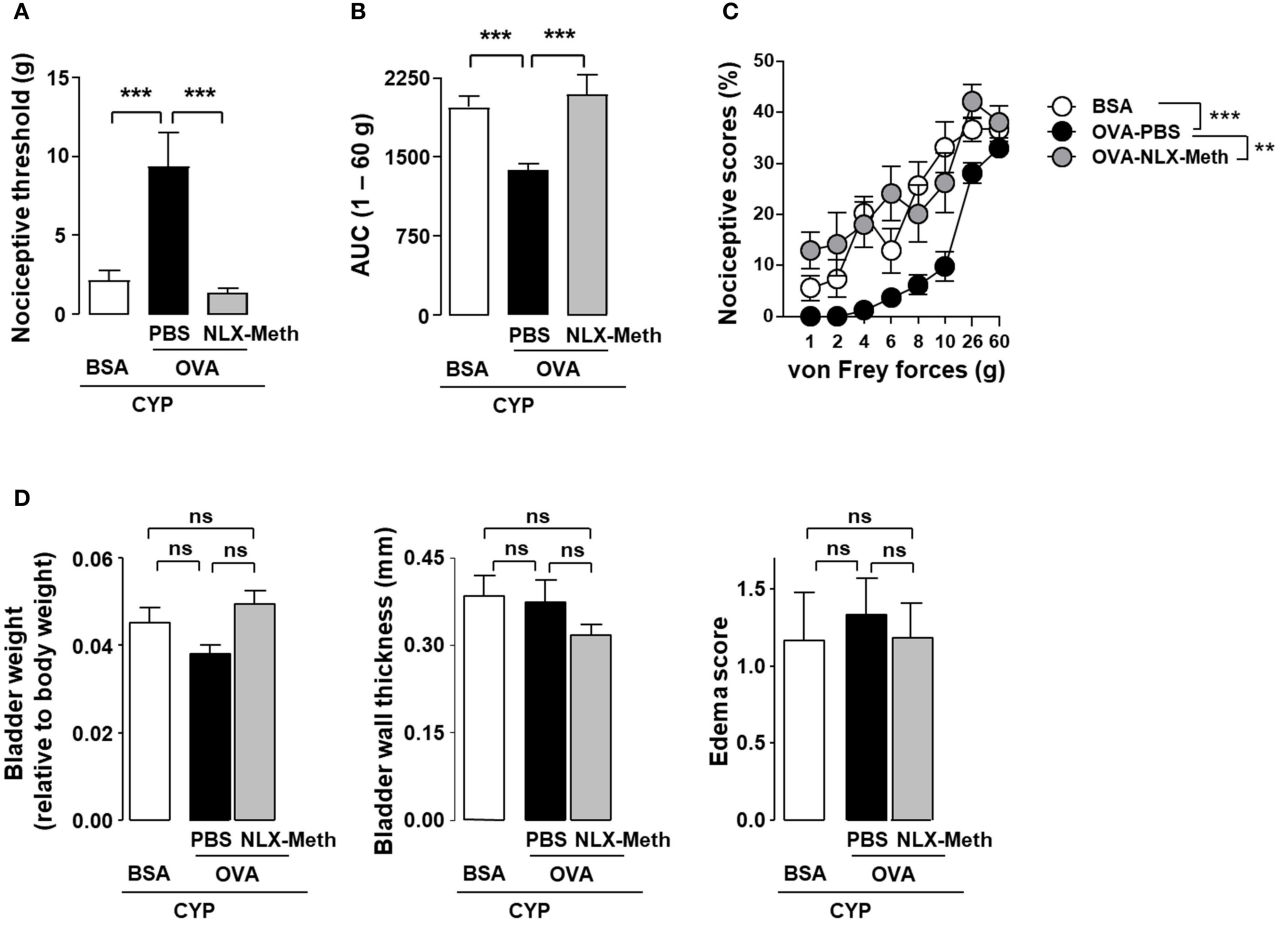
Peripheral opioid receptor blockade inhibits the analgesic effect of OVA instillation in OVA-immunized rats with cystitis. Rats were primed by subcutaneous injection of OVA emulsified in CFA and then boosted 2 weeks later with the same amount of OVA emulsified in IFA. One month after the last immunization, chronic cystitis was induced as described above. OVA-immunized rats were intravesically instilled with either OVA or irrelevant control BSA antigen on days 3, 6, and 7. CYP-induced allodynia **(A)** and hyperalgesia **(B)** was assessed at day 10, in OVA-immunized rats intravesically instilled with either irrelevant control BSA antigen (*n* = 6) or cognate OVA antigen. In addition, in OVA-instilled rats, PBS (*n* = 9) or naloxone-methiodide (NLX-Meth, *n* = 11) was intraperitoneally injected. Nociceptive scores against von Frey forces from 1 to 60 g used to calculate AUC are shown in **(C)**. Urinary bladder inflammation **(D)** was assessed at day 10 by measuring bladder weight (left panel), wall thickness (middle panel) and edema scores (right panel). Data are means values ± SEM. ns, no significant; **p < 0.01 and ***p < 0.001.

The anti-nociceptive effect of antigen secondary challenge was first assessed by the use of the classical ovalbumin (OVA) antigen model. Rats were immunized with OVA and 1 month after the last immunization with OVA, cystitis was induced by CYP repeated injections (26). Animals were then intravesically instilled with the cognate antigen OVA or irrelevant control antigen BSA (Figure 1). As shown in Figure 2, as compared to basal values, CYP injection induced an increase in nociceptive responses in BSA-challenged rats whereas in OVA-challenged rat the pain response stay in basal level (Figures 2A,B). Furthermore, OVA-immunized rats locally injected with OVA displayed a significant reduction of vesical pain as compared to those injected with the irrelevant control antigen BSA (Figures 2C–F). No significant difference was observed for bladder inflammation between OVA- and BSA-challenged rats (Figure 2G). Immunohistochemistry revealed a widespread T lymphocyte mucosal tissue infiltration associated with OVA local intravesical antigen recall (Figures 2H,I). In contrast, very rare T lymphocyte staining was present into bladder mucosa of BSA-challenged rats (Figures 2H,I). The analgesia accompanying the recruitment of T lymphocytes induced by intravesical OVA was reversed by naloxone-methiodide (a general opioid receptor antagonist unable to cross blood-brain barrier) (Figures 3A–C). This indicates that vesical pain inhibition was dependent on the local release of endogenous opioids. The administration of naloxone methiodide 30 min before pain assessment had no effect on urinary bladder inflammation (Figure 3D).

### Analgesic Effects of BCG and Tetanus Toxoid Vaccines in Interstitial Cystitis/Bladder Pain Syndrome Model

**Figure 4:**
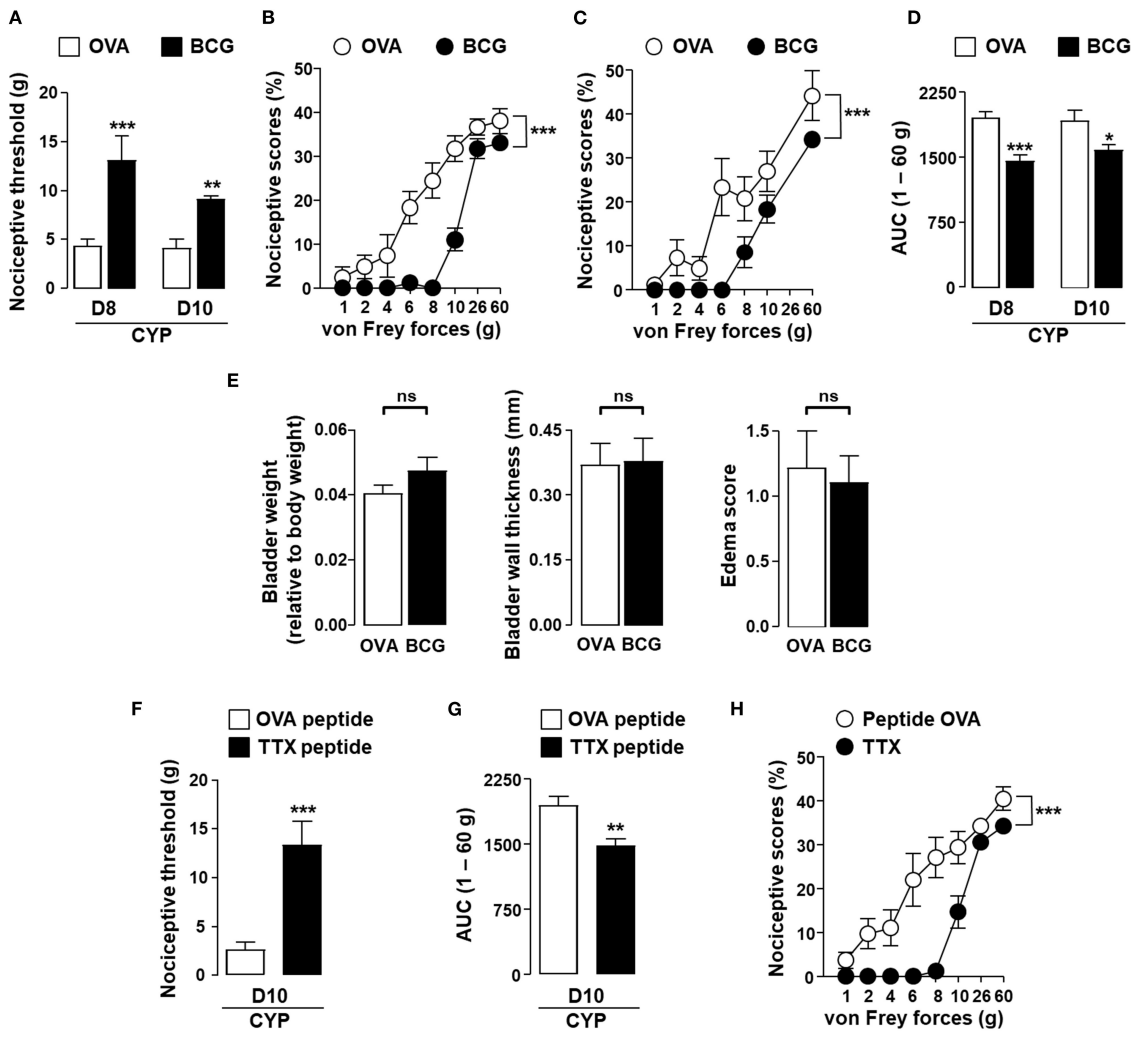
*In situ* instillation of vaccine antigens alleviates bladder pain associated with chronic cystitis in vaccinated rats. Rats were vaccinated by subcutaneous injection of BCG or tetanus toxoid (TTX) as indicated and boosted 2 weeks later with the same amount of vaccine antigens. One month after the last immunization, chronic cystitis was induced as described above. Rats vaccinated against BCG were intravesically instilled with either irrelevant control antigen OVA (white symbols) or BCG (black symbols) on days 3, 6, and 7. **(A)** Nociceptive threshold defined as the von Frey force in grams at which a first score of at least one was obtained (allodynia). **(B,C)** Nociceptive scores against von Frey forces at days 8 **(B)** and 10 **(C)** after CYP treatment. **(D)** Area under the curve (AUC) calculated by plotting individual nociceptive score against von Frey forces from 1 to 60 g. **(E)** Urinary bladder inflammation assessed at day 10 in rats vaccinated with BCG. Rats vaccinated against tetanus toxoid were intravesically instilled with an equimolar mixture of the two immune-dominant TTX-derived peptides (black symbols) or irrelevant control OVA (323–339) peptide (white symbols) on days 3, 6, and 7. Nociceptive threshold **(F)** and AUC 1–60 g **(G)** calculated from nociceptive scores against von Frey forces from 1 to 60 g **(H)** were assessed at day 10 after CYP treatment. Data represent mean values ± SEM (*n* = 9/group). ns, no significant; *p < 0.05, **p < 0.01, and ***p < 0.001.

In order to establish that the results observed with OVA could be extended to a general vaccine strategy, we investigated whether BCG or tetanus toxoid vaccine could alleviate vesical pain in our model. In rats vaccinated against BCG, local challenge with BCG resulted in a decrease in CYP-induced allodynia and hyperalgesia as compared to irrelevant control antigen OVA (Figures 4A–D). BCG instillation had no effect on vesical sensitivity of vaccinated non-CYP-treated rats (data not shown). It is noteworthy that BCG had no effect on inflammatory parameters in the bladder of CYP-induced cystitic rats (Figure 4E). In agreement with the results obtained with BCG, intravesical instillation of universal immunodominant tetanus-derived peptides in rats previously vaccinated with tetanus toxoid significantly reduced CYP-induced bladder pain as compared to irrelevant control OVA peptide (Figures 4F–H).

## Discussion

Current treatments of IC/BPS have controversial efficacy, particularly on pain symptoms. Therefore, development of new therapies is needed. In the present study, we demonstrated that intravesical antigens application on previously vaccinated rats, significantly relieved from CYP-induced bladder pain. These results point out the potential use of vaccine therapy for pain management in IC/BPS patients on the basis of any commonly used vaccine.

As previously reported for intestinal inflammation (23), in vaccinated animals, the induction of a secondary immune response by instillation of vaccine antigens results in the recruitment of T lymphocytes into the mucosa and organ pain relief. In the bladder, we report that this analgesic effect occurs in a naloxone methiodide-dependent fashion indicating that vesical pain inhibition could be due to the local release of endogenous opioids by T lymphocytes accumulating into the mucosa, thereby increasing the constitutive tonic activation of peripheral opioid receptors on sensory neurons (34). Because primed T lymphocytes release opioids upon antigen stimulation (17), the anti-nociceptive effect of antigen recall in vaccinated individuals probably would need regular antigen injections to be maintained.

In humans, the use of intravesical BCG as a potent immune-stimulator for IC/BPS was first reported by Zeidman et al. (35). Several other non-randomized clinical trials including highly heterogeneous patients with IC/BPS resulted in disparate clinical outcomes (36–38). Most importantly, the vaccination status of the patients was never mentioned or investigated. In order to be used in patients, a vaccine recall that would insure a functional vaccination status would be necessary before applying intravesical BCG. Possible correlations between vaccination status and symptomatic responses to BCG treatments could then be investigated. Although BCG-antigen recall therapy has been used for years in the treatment of bladder cancer, its exact mechanism of action is largely unknown. It has been suggested that intravesical instillation of BCG leads to stimulation of the type 1 helper T-cell response (37) and sometimes to type 1 and type 2 helper T-lymphocytes (39), all of these T cell subsets producing endogenous opioids (11, 17, 40).

Our results also demonstrated that the strategy of antigen instillation into cystitis bladder did not modify inflammatory parameters. This is in agreement with the results obtained in patients IC/BPS receiving intravesical BCG that report no worsening of interstitial cystitis symptoms (37).

Taking together, our results suggest that local induction of a secondary T cell response to vaccine antigens, against which the immune system has been primed, prevents from vesical pain by the release of endogenous opioids and further activation of peripheral opioid receptors. These results tend to support that vaccinal approaches represent a promising strategy to manage visceral pain occurring in IC/BPS. To confirm our findings in humans, clinical trial comparing BCG therapy with or without previous BCG vaccination should be set up. Considering the universality of BCG or tetanus vaccination programs worldwide, our results open new therapeutic avenues in the management of IC/BPS.

## Data Availability Statement

The original contributions presented in the study are included in the article/supplementary material, further inquiries can be directed to the corresponding author/s.

## Ethics Statement

The animal study was reviewed and approved by French Animal Ethical Committee application number CEEA-122-2014-28 and APAFIS#16506-2018082411278474.

## Author Contributions

NV, PL, SC, and GD elaborated the study concept. CA, LB, and CB performed and planned the experiments. CA, LB, SC, and GD designed and analyzed the data. GD, CA, SC, and NV wrote the manuscript. GD and PL supervised the study. All authors contributed to the article and approved the submitted version.

## Conflict of Interest

The authors declare that the research was conducted in the absence of any commercial or financial relationships that could be construed as a potential conflict of interest.
